# Hispanic and Immigrant Paradoxes in U.S. Breast Cancer Mortality: Impact of Neighborhood Poverty and Hispanic Density

**DOI:** 10.3390/ijerph13121238

**Published:** 2016-12-14

**Authors:** Sandi L. Pruitt, Jasmin A. Tiro, Lei Xuan, Simon J. Craddock Lee

**Affiliations:** 1 Department of Clinical Sciences, University of Texas Southwestern Medical Center, Dallas, TX 75390, USA; jasmin.tiro@utsouthwestern.edu (J.A.T.); lei.xuan@utsouthwestern.edu (L.X.); simoncraddock.lee@utsouthwestern.edu (S.J.C.L.); 2 Harold C. Simmons Comprehensive Cancer Center, Dallas, TX 75235, USA

**Keywords:** breast cancer, disparities, inequality, survival, Hispanic, neighborhoods, immigration, poverty, ethnic enclave

## Abstract

To test the Hispanic and Immigrant Paradoxes—i.e., survival advantages despite a worse risk factor profile—and the modifying role of neighborhood context, we examined associations between patient ethnicity, birthplace, neighborhood Hispanic density and neighborhood poverty among 166,254 female breast cancer patients diagnosed 1995–2009 in Texas, U.S. Of all, 79.9% were non-Hispanic White, 15.8% Hispanic U.S.-born, and 4.2% Hispanic foreign-born. We imputed birthplace for the 60.7% of Hispanics missing birthplace data using multiple imputation. Shared frailty Cox proportional hazard models (patients nested within census tracts) adjusted for age, diagnosis year, stage, grade, histology, urban/rural residence, and local mammography capacity. Whites (vs. U.S.-born Hispanics) had increased all-cause and breast cancer mortality. Foreign-born (vs. U.S.-born) Hispanics had increased all-cause and breast cancer mortality. Living in higher Hispanic density neighborhoods was generally associated with increased mortality, although associations differed slightly in magnitude and significance by ethnicity, birthplace, and neighborhood poverty. We found no evidence of an Immigrant Paradox and some evidence of a Hispanic Paradox where protective effects were limited to U.S.-born Hispanics. Contrary to prior studies, foreign birthplace and residence in higher Hispanic density neighborhoods were associated with increased mortality. More research on intersections between ethnicity, birthplace and neighborhood context are needed.

## 1. Introduction

Among Hispanic women in the U.S., breast cancer is the most common cancer and leading cause of cancer death [1]. Despite a worse risk factor profile, Hispanics overall demonstrate better or equivalent survival outcomes compared to non-Hispanic Whites [2,3,4]. This well-documented and persistent epidemiologic phenomenon is referred to as the Hispanic Paradox [5]. For breast cancer, data generally, but not always, support the Hispanic paradox [6,7]. For example, we recently found that among urban breast cancer patients in Texas, Hispanic women had lower all-cause, but not breast cancer-specific, mortality compared to non-Hispanic White women [8]. 

Researchers posit that the Hispanic Paradox reflects social and cultural advantages rather than biological [4,9,10]. Two commonly assessed proxy measures of exposure to Hispanic culture, patient birthplace (U.S. vs. foreign) and residence in a neighborhood with other Hispanics, have been associated with mortality. Foreign-born, compared to U.S.-born Hispanics, often have lower cancer mortality, despite worse risk factor profiles [11,12,13]. The protective effect of foreign birthplace is referred to as the Immigrant Paradox. A California study showed that Hispanic foreign-born breast cancer patients (vs. U.S.-born) had lower risk of all-cause (but not breast cancer) death after adjustment for covariates [11]. However, evidence for or against an Immigrant Paradox in breast cancer is inconclusive given limited research to date.

The percentage of Hispanics in a neighborhood (i.e., neighborhood Hispanic density) may also convey and/or reflect cultural factors that improve survival. The protective advantages of living in high Hispanic density neighborhoods have been referred to as barrio effects [14]. Hispanics with cancer living in neighborhoods with higher Hispanic density (i.e., ethnic enclaves) may experience a survival advantage [12,13]. However, neighborhoods with large Hispanic and/or foreign-born populations also experience disproportionate socioeconomic deprivation, including concentrated poverty [15,16]. For cancer patients and others, residence in a socioeconomically deprived neighborhood is associated with adverse outcomes, including higher mortality [17,18]. Thus, to elucidate the impact of neighborhood Hispanic density, neighborhood socioeconomic status must also be accounted for in the analytic modeling strategy [11,19].

A growing consensus in cancer inequality research—including the geospatial and multilevel variation literature—encourages an intersectionality approach wherein multiple indicators of social status are concurrently examined [20,21,22]. For any given individual or population, meaning and consequences of various cultural and social categories (e.g., ethnicity and socioeconomic status) are often the cause and consequence of macro-level social policies (e.g., residential segregation) and therefore are highly interdependent. Thus, an intersectional approach—one that examines multiple social indicators simultaneously—is needed to elucidate the extent to which Hispanic differences in cancer mortality are confounded or modified by the unmeasured role of culture [20,21]. However, to date, research incorporating proxy measures of exposure to Hispanic culture is often limited to a single ethnic group (i.e., Hispanics only) [11,13,23,24], and has not tested the Hispanic paradox in cancer (i.e., compared outcomes between Hispanics and Whites). Thus, prior work has precluded more nuanced examinations at the intersections of social statuses across (e.g., Hispanic paradox) and within (e.g., foreign- vs. U.S.-born) ethnicities. 

This gap in the literature may be partly explained by limitations in U.S. cancer registry data. Birthplace is frequently missing [25,26,27,28]. Dropping patients missing data reduces generalizability and introduces bias because patients missing birthplace are more likely to be survivors, English-speakers, diagnosed at earlier stages, and U.S.-born [25,26,27,28,29,30]. Thus, imputation of missing data is required to accurately assess outcomes [28,31,32]. Neighborhood-level variables such as ethnic density or poverty may be appended to registry data using geographic identifiers that are commonly available (e.g., census tract, latitude, longitude). Since it requires additional administrative review and specialized geographic information system (GIS) software [33,34], this approach remains underutilized.

We fill these evidence gaps and take an intersectional, multilevel approach by examining association of patient birthplace and residence in a neighborhood with other Hispanics (Hispanic culture proxies) on mortality among White and Hispanic women with breast cancer. We hypothesized the following:

1. Whites have increased mortality (Hispanic Paradox) and foreign-born Hispanics have decreased mortality (Immigrant Paradox) compared to U.S.-born Hispanics.
2. Neighborhood Hispanic density moderates ethnic differences in mortality such that living in a neighborhood with more Hispanics is protective for Hispanics (foreign- and U.S.-born) and harmful for Whites.

Given that greater Hispanic density often co-occurs with higher neighborhood poverty and exposure to these two factors differs dramatically among ethnicity/birthplace groups, we also explored the combined effect of both factors on mortality separately for each group.

## 2. Materials and Methods

### 2.1. Sample

We obtained data from the Texas Cancer Registry (TCR), a North American Association of Central Cancer Registries (NAACCR) gold-certified population-based registry. We included female adult (≥18 years old) breast cancer patients diagnosed between 1995–2009, limiting data to non-Hispanic White or Hispanic (of any race) women with a first or only primary cancer with a known diagnosis date. We excluded patients diagnosed at autopsy or death. Using latitude and longitude of residence at diagnosis (TCR geocoded data) we merged year 2000 U.S. Census data provided by Geolytics Inc. (Census 2000, Long Form (SF3), GeoLytics, Inc., East Brunswick, NJ, USA, 2012) and year 2000 mammography machine data obtained from a freedom of information request to the US Food and Drug Administration (FDA). The Institutional Review Boards at UT Southwestern Medical Center (#STU 082012-064) and Texas Department of Health and Social Services (#12-060) approved this study. 

### 2.2. Outcomes

We assessed two outcomes: all-cause and breast cancer-specific mortality as a function of survival time (months between diagnosis and death date) and censored those alive on 31 December 2011. For the breast cancer-specific analysis, we censored deaths from other causes. TCR ascertains vital status and cause of death using annual linkage to multiple vital status data sources. 

### 2.3. Independent Variables

#### 2.3.1. Race/Ethnicity

We measured race/ethnicity as non-Hispanic White (hereinafter “White”) or Hispanic (of any race; 97% were White) using TCR data. Hispanics were defined using the NAACCR Hispanic Identification Algorithm, considered best practice for enhancing Hispanic identification in cancer registry data [35] 

#### 2.3.2. Birthplace

Among Hispanics, 26.8% were U.S.-born, 12.6% were foreign-born, and 60.7% were missing birthplace. To impute missing values, we adapted methods previously described [28,32]. We first randomly split the subsample with birthplace into training (80%) and validation (20%) groups and merged cases missing birthplace to the training group. Using the training group, we imputed birthplace values (1 = foreign-born; 0 = U.S.-born) over 20 iterations [28] to obtain posterior predictive distribution of parameters using the SAS Multiple Imputation procedure (PROC MI with LOGISTIC in the MONOTONE statement (SAS 9.4; SAS Institute Inc., Cary, NC, USA)). For descriptive statistics, 20 imputed values were averaged, with mean >0.5 recoded as 1 and ≤0.5 recoded as 0; in analytic models, values were imputed once for ease of computation.

The imputation model a priori included nine TCR variables (age, stage, Hispanic origin, reporting source, diagnosis year, method of diagnostic confirmation, class of case, tumor grade, and histology) and four U.S. Census variables representing patients’ residential census tract (percent foreign-born, Hispanic, speaks language other than English, and living in poverty). We added tumor histology and the census tract variables to those previously described [28].

Next, we measured model performance using the validation group. Comparing true and imputed birthplace, sensitivity (0.82), specificity (0.84), kappa (0.64) and misclassification rate (0.16) all indicated substantial agreement [36]. Thus, the multiple imputation model accurately estimated birthplace. Finally, we used the full data set (combining training and validation groups) to impute missing birthplace values.

### 2.4. Neighborhood Measures

Following recommendations and our prior practice, we defined neighborhoods as patient residential census tract [8,37]. We examined two continuous neighborhood factors: (1) Hispanic density, measured as census tract percent Hispanic; and (2) Neighborhood poverty, measured as census tract percent living at or below federal poverty level.

### 2.5. Covariates

Patient-level covariates included: age (18–39, 40–49, 50–59, 60–69, 70–79, ≥80); Surveillance, Epidemiology, and End Results Program (SEER) summary stage (in situ, local, regional, distant, unstaged); diagnosis year (1995–1997, 1998–2000, 2001–2003, 2004–2006, 2007–2009); tumor grade (low, high, unknown differentiation); and histology (lobular, ductal, other, unknown).

Neighborhood-level covariates included: census tract-based urban/rural status using year 2000 Rural Urban Commuting Area codes [38,39]; and mammography capacity (poor, adequate, excess) using the two-step floating catchment area (2SFCA) method using methods previously described [39,40,41]. Mammography capacity is included as a proxy for (1) prior mammography screening utilization (not available in TCR) beyond what is captured by stage at diagnosis and (2) availability of healthcare resources.

### 2.6. Analysis

We conducted descriptive analyses separately for the ethnicity/birthplace groups and compared distributions using chi-square and one-way ANOVA. We fitted three Cox Proportional Hazard models for each outcome. To test Hypothesis 1, we fitted univariate models (Model 1) to examine the main effects of ethnicity/birthplace and neighborhood Hispanic density on mortality. We then added these variables and all covariates (Model 2). U.S.-born Hispanics were selected as the referent group to facilitate examination of both the Hispanic Paradox and the Immigrant Paradox. To test Hypothesis 2, we introduced two interaction terms into the adjusted model (neighborhood percent Hispanic* (White/foreign-born Hispanic)) (Model 3). We tested the assumption of proportional hazards using this final model. To qualitatively examine significant (*p* < 0.05) interactions of a continuous and categorical variable, we generated plots of the marginal effect of neighborhood Hispanic density for each ethnicity/birthplace group.

Since poverty is highly correlated with neighborhood Hispanic density, we explored combined effects of neighborhood Hispanic density and neighborhood poverty. We calculated categories of both variables (below vs. above median) due to dramatically different distributions and limited overlap on these variables across the three ethnicity/birthplace groups. We generated a four-category variable (i.e., low vs. high poverty/Hispanic density) for each ethnicity/birthplace group. For each group, we examined the combined variable in univariate (not shown) and adjusted proportional hazard models including all covariates. 

We fitted all hazard models as shared frailty models (i.e., multilevel models including random effects accounting for clustering of patients within their residential census tracts). Descriptive analyses were conducted in SAS 9.4. Shared frailty models were estimated using the coxme program and we tested the assumption of proportional hazards using the cox.zph program in R 3.0.2 [42]. Plots of marginal effects were generated using Stata margins and marginsplot commands after fitting an unadjusted Cox model (Stata/SE 13.1; StataCorp LP, College Station, TX, USA).

## 3. Results

### 3.1. Sample Characteristics

Of 166,254 women (Table 1), 79.9% were White, 15.8% were U.S.-born Hispanics, and 4.2% were foreign-born Hispanics. All characteristics differed between ethnicity/birthplace groups (*p* < 0.05). For example, foreign-born Hispanics lived in neighborhoods with the highest Hispanic density and poverty rate, followed by U.S.-born Hispanics, and finally Whites. The crude proportion of those dying from all causes was highest among foreign-born Hispanics and lowest among U.S.-born Hispanics. The crude proportion dying from breast cancer was highest among foreign-born Hispanics and lowest among Whites.

### 3.2. Hypothesis 1: Mortality Differences by Ethnicity/Birthplace

For all-cause mortality (Table 2), univariate and adjusted models indicated that Whites (Model 2 adjusted hazard ratio (aHR): 1.12; 95% confidence intervals (CI): 1.08–1.15) and foreign-born Hispanics (Model 2 aHR: 1.47; 95% CI: 1.40–1.54) had increased mortality when compared to U.S.-born Hispanics. 

Results differ slightly for breast-cancer mortality (Table 2). Whites (vs. U.S.-born Hispanics) had decreased mortality in the unadjusted model (Model 1 HR: 0.91; 95% CI: 0.87–0.94). However, increased mortality (although marginally significant) was observed in the adjusted model (Model 2 aHR: 1.05; 95% CI: 1.00–1.10). Foreign-born (vs. U.S.-born) Hispanics had increased mortality in both unadjusted and adjusted models (aHR: 1.60; 95% CI: 1.50–1.70). 

### 3.3. Hypothesis 2: Effect of Neighborhood Hispanic Density by Ethnicity/Birthplace 

**Figure 1:**
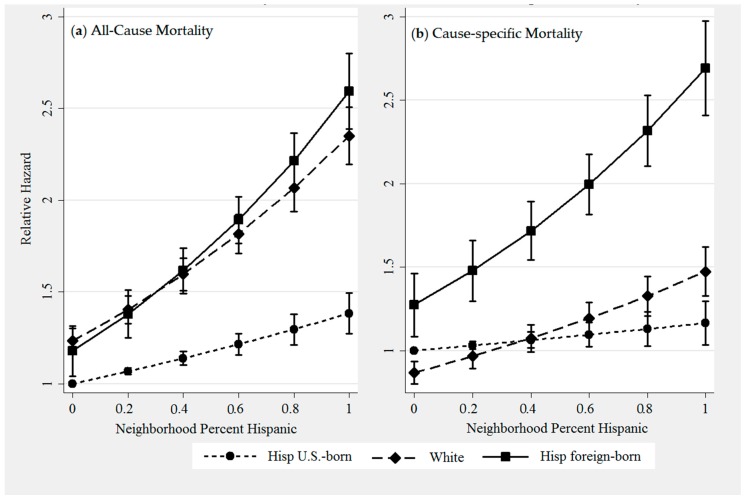
Predicted all-cause (**a**) and breast cancer-specific (**b**) relative hazard and 95% confidence intervals by neighborhood percent Hispanic for foreign-born Hispanics, U.S.-born Hispanics and Whites. Margins represent estimates generated from unadjusted Cox Proportional hazard models.

In Models 1 and 2, women living in higher Hispanic density neighborhoods had increased mortality in unadjusted and adjusted models for both outcomes. However, Model 3 demonstrates that interactions between patient ethnicity/birthplace and neighborhood Hispanic density were statistically significant (*p* < 0.05). Figure 1 displays plots of margins including 95% confidence intervals. While the direction of the association between density and mortality was positive (higher density associated with increased mortality), the magnitude of the association differs across groups and by cause of death. The association of increased Hispanic density with higher *all-cause* mortality is strongest among foreign-born Hispanics and Whites (panel 1a), whereas its association with higher *breast-cancer* mortality is strongest among foreign-born Hispanics (panel 1b). Notably, the association of Hispanic density on mortality for Whites differs greatly depending on cause of death. The assumption of proportional hazards was not violated for any of the primary variables of interest (ethnicity, birthplace, neighborhood Hispanic density) in the final model (Model 3). 

Figure 1 also reflects the Model 2 finding that mortality differences between groups persist regardless of neighborhood Hispanic density. For example, panel 1a demonstrates the all-cause mortality advantage of U.S.-born Hispanics compared to other groups. Conversely, panel 1b demonstrates the breast-cancer mortality.

### 3.4. Combined Effects of Neighborhood Hispanic Density and Neighborhood Poverty 

For Whites, living in high Hispanic density and/or high poverty neighborhoods was consistently associated with higher mortality. Living in any combination of a high Hispanic density neighborhood or high poverty neighborhood was associated with increased all-cause and breast-cancer mortality, compared to those living in low poverty and low Hispanic density neighborhoods (Table 3).

For U.S.-born Hispanics, there appeared to be little effect of living in high Hispanic density and/or high poverty neighborhoods. The one exception was increased all-cause mortality (aHR: 1.19; 95% CI: 1.09–1.30) among those living in low Hispanic/high poverty neighborhoods, compared to low Hispanic/low poverty neighborhoods.

Foreign-born Hispanics, unlike U.S.-born Hispanics, experienced higher all-cause and breast cancer mortality when exposed to higher neighborhood Hispanic density and/or poverty. Foreign-born Hispanics living in high poverty neighborhoods, regardless of neighborhood Hispanic density, had higher mortality on both outcomes. For example, those in high Hispanic/high poverty (vs. low Hispanic/low poverty) neighborhoods had increased all-cause (aHR: 1.39; 95% CI: 1.25–1.53) and breast-cancer (aHR: 1.33; 95% CI: 1.17–1.51) mortality. In contrast, living in a high Hispanic and low poverty neighborhood was associated with higher all-cause mortality, it was not associated with breast-cancer mortality.

## 4. Discussion

We examined the association of mortality with two proxy indicators of Hispanic culture—patient birthplace and residence in a neighborhood with other Hispanics—among White and Hispanic women with breast cancer in Texas using an intersectional, multilevel approach. We also explored and disentangled the combined effects of two interrelated neighborhood characteristics, neighborhood Hispanic density and neighborhood poverty. Our findings largely refute the Hispanic and the Immigrant Paradoxes, and add novel insights regarding the interactions of ethnicity, birthplace, neighborhood poverty, and neighborhood ethnic density—factors largely studied in isolation. 

### 4.1. Mortality by Ethnicity/Birthplace

Hypothesis 1 (Whites have increased and foreign-born Hispanics have decreased mortality compared to U.S.-born Hispanics) was only partially supported. 

Findings regarding U.S.-born Hispanics versus Whites largely uphold the concept of a Hispanic paradox. The hypothesized White disadvantage compared to U.S. Hispanics was observed for both all-cause and breast cancer-specific mortality. However, the direction of the association flipped between unadjusted and adjusted breast cancer models and the effect size indicates a small, but significant, White disadvantage in breast cancer mortality (aHR = 1.05). Even a small difference in mortality can still be considered paradoxical, however, as even similar outcomes between Hispanics and Whites are inconsistent with the wide inequalities in risk factor profiles [43,44]. Several researchers have posited that Hispanic culture endorses healthy behaviors and provides social ties that reduce mortality. All-cause survival (vs. breast cancer survival) may be more sensitive to these beneficial behavioral and social influences conveyed by Hispanic culture. For example, all-cause mortality includes deaths from cardiovascular disease, which is closely linked with behavioral factors such as diet. In contrast, breast cancer-specific mortality is likely more influenced by suboptimal diagnosis and treatment experiences of Hispanics. Compared to Whites, Hispanic breast cancer patients have later stage at diagnosis, are more likely to experience treatment delays, and are less likely to receive therapies meeting the recommended standard of care [7,45]. 

Contrary to hypotheses, foreign-born (vs. U.S.-born) Hispanics had higher mortality, which contradicts the Immigrant Paradox observed in prior studies [4,46,47,48,49,50]. Many prior studies documenting a foreign-born advantage in cancer survival were conducted among general populations and did not use registry data of patients with confirmed cancer. Thus, they failed to account for the lower incidence of many cancers (e.g., breast, lung, prostate, colorectal) experienced by the foreign-born and were unable to control for critical prognostic factors (e.g., stage at diagnosis) that differ by birthplace. In contrast, studies using cancer registry data are conducted only among cancer patients and can adjust for numerous covariates. Overall, cancer registry studies on the Hispanic paradox have demonstrated mixed findings [12,13,23,24,28,32,51]. In a registry analysis of breast cancer specifically, a California study of female Hispanic patients found a foreign-born advantage (vs. U.S.-born) in all-cause survival but equivalent breast cancer survival [11]. Mixed findings across studies may result from unmeasured differences within the foreign-born population—e.g., country of origin, documentation, and citizenship status. Furthermore, direct comparisons across studies are challenged by use of varied referent groups and strategies for handling missing birthplace data. White is often the de facto referent group; the rationale for this choice is implicit. We urge researchers to carefully consider alternative referent groups as appropriate to examine their research questions [52]. In our study, we used U.S.-born Hispanics as our referent group to facilitate testing the Immigrant paradox, which requires comparing foreign- to U.S.-born Hispanics.

Researchers have posited that culture is a positive force driving longer life expectancy for Hispanics, particularly the foreign-born. Our findings suggest some alternative interpretations. First, debate regarding the role of selection effects and bias in death ascertainment continues, and has not been extensively studied with cancer mortality [51]. We cannot rule out such biases in our study. Second, from an intersectional perspective, the effects of Hispanic culture cannot be isolated from other structural factors [53]. Beneficial effects are plausible when Hispanic culture is considered as an independent construct operating at an individual or intra-ethnic level. For example, evidence suggests health-affirming behavioral risk profiles and social networks [4,54,55,56,57,58,59] among Hispanics and the foreign-born. However, the relative beneficial contribution of these factors may be undermined by disadvantageous structural factors, including discrimination, immigration policies, constrained socioeconomic opportunities, and limited availability or access to resources, including cancer care [53]. Thus, the foreign-born disadvantage observed herein may result from such factors, which were not fully adjusted for here. These may include lower mammography adherence, later stage at diagnosis, and lower odds of having health insurance and a source of usual care among foreign- versus U.S.-born Hispanic women [2,60,61,62,63].

In sensitivity analyses (not shown), we dropped all Hispanics missing birthplace (*n* = 20,247), a common approach to missing data in prior studies [25,28]. Two differences emerged. First, both Whites and foreign-born Hispanics had lower all-cause mortality compared to U.S.-born Hispanics (vs. increased mortality observed when using imputed data). Second, Whites and foreign-born Hispanics had lower breast cancer mortality compared to U.S.-born Hispanics (vs. imputed data analyses showing similar results for Whites and increased mortality for foreign-born Hispanics). 

The reversal of findings upon listwise deletion of Hispanics missing birthplace was an expected finding for several reasons. First, as described in the Introduction, multiple factors are well-known to be associated with missing birthplace [25,26,27,28,29,30]. We confirmed this in our study by comparing distribution of all study outcomes and covariates for Hispanics with birthplace versus those missing birthplace. Those missing birthplace were different (all *p* < 0.001) on all measured covariates and outcomes except tumor histology [data not shown]. The most notable difference, as documented in prior studies [25,26,27,28,29,30], is that those missing birthplace are more likely to be alive (because death certificates provide the primary source of information about patient birthplace.) Since those missing birthplace are more likely to be alive and also more likely to be U.S. citizens [25,26,27,28,29,30], dropping those patients from our sensitivity analysis resulted in an erroneously low mortality for U.S.-born citizens. Thus, sensitivity analyses showed nearly the polar opposite results from our primary results. The same reversal of findings was also documented in a prior SEER cancer registry. In that study, as in ours, the apparent survival advantage of foreign- vs. U.S.-born Hispanic patients when using listwise deletion of those missing birthplace was reversed or nullified when birthplace was imputed [28]. These findings suggest that investigators proceed cautiously when considering dropping patients missing birthplace from their analysis, particularly when the missing rate is high, due to the potential for significant bias.

### 4.2. Effect of Neighborhood Hispanic Density by Ethnicity/Birthplace 

Hypothesis 2, that neighborhood Hispanic density moderates ethnic differences in mortality was supported; however, not as hypothesized. While Hispanic density significantly moderated ethnic differences in survival, such that increasing density was consistently associated with increased mortality for Whites, the direction of the association for Hispanics differed from prior studies. While interaction terms indicated that the slope of the association was statistically different between the three groups, we observed no protective effect of Hispanic density among U.S.- or foreign-born Hispanics as hypothesized. These findings contribute to the growing evidence that ethnically dense neighborhoods are not uniformly advantageous for all healthy behaviors and outcomes, even for co-ethnic (same ethnicity) residents [16,19].

### 4.3. Combined Effects of Neighborhood Hispanic Density and Poverty

When exploring the combined effect of neighborhood Hispanic density and poverty, we found different patterns of association by ethnicity/birthplace. Perhaps unsurprisingly, Whites living in any combination of high poverty and/or high Hispanic density neighborhoods had increased mortality from both causes. Similarly, foreign-born Hispanics generally experienced increased mortality from exposure to high poverty and/or high Hispanic density neighborhoods. In contrast, all but one combination of high Hispanic density and/or poverty was significantly associated with increased mortality for U.S.-born Hispanics. We observed higher mortality among U.S.-born Hispanics living in high poverty neighborhoods, but only for those in low Hispanic density neighborhoods, and this was only observed for all-cause mortality. Findings suggest that for U.S.-born Hispanics, neighborhood ethnic and socioeconomic context may not be as deleterious as for other population groups; or it may indicate that such neighborhood exposures provide both advantages and disadvantages for U.S.-born Hispanics.

Findings support prior studies indicating that birthplace, neighborhood socioeconomic status, and geographic region may moderate effects of neighborhood ethnic density on cancer outcomes [11,12,13,23,24,64]. We wholeheartedly agree with Gomez et al.’s conclusion that the interactions of these factors are “exceedingly complex” [24]. Future research from other cancer registries with large Hispanic populations—beyond Texas, California, and SEER registries examined in most existing U.S. studies—may shed light on mixed results to date.

### 4.4. Limitations

Our study is subject to limitations. First, although missing birthplace data in registries is a well-known phenomenon [25,27,29,30], missingness (60.7% of Hispanics) was high. Resulting misclassification from our imputation could have biased effect estimates. Birthplace is a frequently overlooked indicator of culture, in part due to missing data. Future research is needed to widely apply, validate, and improve upon the imputation method herein [28] or the alternative imputation approach using patient social security number [11,61]. Breast cancer–specific analyses may be biased due to inaccuracies in cause-of-death coding. Data were unavailable for several prognostic factors (e.g., performance status, treatment) as well as length of neighborhood residence and data cannot be used to infer causality. Finally, our results may not be generalizable to patients outside of Texas, where Hispanics are predominantly White (95%) and most foreign-born are from Mexico (84%) [65]. Other U.S. regions may have Hispanic populations who differ by race, country of origin, nativity status, settlement patterns, or length of time in the U.S.

## 5. Conclusions

We examined Hispanic and Immigrant Paradoxes among breast cancer patients using an intersectional, multilevel approach with two novel proxy indicators of exposure to Hispanic culture. While we provide some evidence of a Hispanic Paradox, the protective effect of Hispanic ethnicity was entirely limited to U.S.-born Hispanic women. We found no evidence of an Immigrant Paradox. Neighborhoods with higher Hispanic density were generally associated with higher mortality but associations differed by patient ethnicity and birthplace. Our results raise important questions about the nature of causal mechanisms underlying ethnic differences in health, including circumstances in which cultural factors may exacerbate or ameliorate these differences.

Results demonstrating differences in mortality by cause of death, ethnicity, birthplace, and neighborhood lend additional support to the call for intersectional research [20]. Given increasing diversity and increasing geographic dispersion of the U.S. Hispanic population, such approaches will be critical for the identification of subpopulations at increased risk.

## Figures and Tables

**Table 1:** Characteristics by ethnicity and birthplace of women diagnosed with breast cancer in Texas, 1995–2009 (*n* = 166,254).

Characteristic	White *n* = 132,870 (79.9%)	Hispanic U.S.-Born ^a^ *n* = 26,328 (15.8%)	Hispanic Foreign-Born ^a^ *n* = 7056 (4.2%)	*p* (Chi^2^)
	%	%	%	
Diagnosis Year				
1995 < year ≤ 1997	18.0	14.0	16.3	*p* < 0.001
1997 < year ≤ 2000	20.3	17.2	19.4	
2000 < year ≤ 2003	20.8	19.8	19.3	
2003 < year ≤ 2006	19.8	22.5	21.9	
2006 < year ≤ 2009	21.1	26.5	23.2	
Age				
Age < 40	4.6	10.1	12.2	*p* < 0.001
40 ≤ Age < 50	17.4	25.8	25.2	
50 ≤ Age < 60	24.7	26.2	24.9	
60 ≤ Age < 70	23.6	19.7	19.2	
70 ≤ Age < 80	19.5	13.2	12.0	
Age ≥ 80	10.3	5.1	6.5	
Stage				
In situ	15.9	14.5	10.7	*p* < 0.001
Localized	50.0	43.9	39.6	
Regional	24.8	31.1	35.1	
Distant	4.1	4.9	7.2	
Unstaged	5.2	5.5	7.3	
Grade				
Low	49.7	40.9	38.5	*p* < 0.001
High	31.9	40.2	39.7	
Unknown	18.4	18.9	21.8	
Histology				
Ductal	68.9	68.8	69.8	*p* < 0.001
Lobular	8.0	6.6	6.0	
Other	21.7	23.0	22.2	
Unknown	1.4	1.7	1.9	
Urban/rural status				
Urban	78.3	85.0	88.3	*p* < 0.001
Rural	21.7	15.0	11.7	
Mammography Capacity				
Adequate	82.7	81.1	79.1	*p* < 0.001
Excess capacity	3.9	3.0	2.8	
Poor	13.4	15.9	18.2	
All-Cause Death				
Alive	71.3	76.6	64.8	*p* < 0.001
Deceased	28.7	23.4	35.2	
Breast-Cancer Death				
Alive	89.1	88.1	79.1	*p* < 0.001
Deceased	10.9	11.9	20.9	
Neighborhood Characteristics	IQR	IQR	IQR	*p* (one-way anova F-test)
Percent Hispanic	6–-24	26–86	34–91	*p* < 0.001
Percent Poverty	5–15	9–-28	11–32	*p* < 0.001

^a^ Missing birthplace values were imputed. SD = standard deviation; IQR = inter-quartile range; *n* = number.

**Table 2:** Hazard ratios and 95% confidence intervals for the association of patient ethnicity and birthplace and neighborhood percent Hispanic on breast-cancer and all-cause mortality among women diagnosed with breast cancer in Texas, 1995–2009 (*n* = 166,254).

**All-Cause Mortality**			
**Patient and Neighborhood Characteristics**	**Unadjusted Univariate Model 1**	**Model 2**	**Model 3**
	**HR (95% CI)**	**aHR (95% CI)**	**aHR (95% CI)**
Patient Ethnicity and Birthplace			
Hispanic-U.S.-born	1	1	1
White	1.24 (1.21–1.28)	1.12 (1.08–1.15)	1.03 (0.99–1.07)
Hispanic-foreign-born	1.64 (1.57–1.72)	1.47 (1.40–1.54)	1.21 (1.12–1.31)
Neighborhood % Hispanic ^a^	1.45 (1.39–1.51)	1.22 (1.16–1.24)	0.95 (0.87–1.03)
Interaction of Neighborhood % Hispanic ^a^ * Hispanic-U.S.-born	-	-	1
Interaction of Neighborhood % Hispanic ^a^ * White	-	-	1.37 (1.24–1.51)
Interaction of Neighborhood % Hispanic ^a^ * Hispanic foreign-born	-	-	1.69 (1.44–1.98)
**Breast Cancer-Specific Mortality**			
**Patient and Neighborhood Characteristics**	**Unadjusted Univariate Model 1**	**Model 2**	**Model 3**
	**HR (95% CI)**	**aHR (95% CI)**	**aHR (95% CI)**
Patient Ethnicity and Birthplace			
Hispanic-U.S.-born	1	1	1
White	0.91 (0.87–0.94)	1.05 (1.00–1.10)	0.97 (0.92–1.02)
Hispanic-foreign-born	1.90 (1.79–2.03)	1.60 (1.50–1.70)	1.28 (1.16–1.42)
Neighborhood % Hispanic ^a^	1.75 (1.66–1.85)	1.19 (1.11–1.28)	0.90 (0.80–1.01)
Interaction of Neighborhood % Hispanic ^a^ * Hispanic-U.S.-born	-	-	1
Interaction of Neighborhood % Hispanic ^a^ * White	-	-	1.43 (1.23–1.65)
Interaction of Neighborhood % Hispanic ^a^ * Hispanic-foreign-born	-	-	1.84 (1.49–2.28)

^a^ Neighborhood percent Hispanic population was centered at the mean. HR = hazard ratio; aHR = adjusted hazard ratio; CI: confidence interval. Models 2 and 3 include age, sex, tumor grade, tumor stage, year of diagnosis, tumor histology, urban/rural status, and mammography capacity. Dashes (–) indicate that the variables were not entered into the model.

**Table 3:** Adjusted hazard ratios and 95% confidence intervals for the association of ethnicity/birthplace group-specific categories of neighborhood percent Hispanic and neighborhood percent poverty on all-cause and breast cancer mortality by patient ethnicity and birthplace among women diagnosed with breast cancer in Texas, 1995–2009 (*n* = 166,254).

Neighborhood Characteristics	White	Hispanic-U.S.-Born	Hispanic-Foreign-Born
	aHR (95% CI)	aHR (95% CI)	aHR (95% CI)
**All-Cause Mortality**			
Low Hispanic/Low poverty	1	1	1
Low Hispanic/High poverty	1.21 (1.16–1.25)	1.19 (1.09–1.30)	1.18 (1.01–1.38)
High Hispanic/Low poverty	1.08 (1.04–1.12)	0.96 (0.87–1.06)	1.19 (1.01–1.41)
High Hispanic/High poverty	1.25 (1.21–1.28)	1.03 (0.97–1.10)	1.39 (1.25–1.53)
**Breast Cancer Mortality**			
Low Hispanic/Low poverty	1	1	1
Low Hispanic/High poverty	1.19 (1.12–1.26)	1.09 (0.96–1.23)	1.22 (1.00–1.48)
High Hispanic/Low poverty	1.09 (1.03–1.15)	0.88 (0.77–1.02)	1.20 (0.97–1.47)
High Hispanic/High poverty	1.24 (1.18–1.30)	0.97 (0.89–1.05)	1.33 (1.17–1.51)

Models are adjusted for age, sex, tumor grade, tumor stage, year of diagnosis, tumor histology, urban/rural status, and mammography capacity.
